# Assessment of real-time electrocardiogram effects on interpretation quality by emergency physicians

**DOI:** 10.1186/s12909-023-04670-x

**Published:** 2023-09-18

**Authors:** Alice Perrichot, Pradeebane Vaittinada Ayar, Pierre Taboulet, Christophe Choquet, Matthieu Gay, Enrique Casalino, Philippe Gabriel Steg, Sonja Curac, Prabakar Vaittinada Ayar

**Affiliations:** 1 Emergency Department, Beaujon Hospital AP-HP, Clichy, France; 2 Laboratoire des Sciences du Climat et l’Environnement (LSCE-IPSL), CNRS/CEA/UVSQ, UMR8212, Université Paris-Saclay, Gif-sur-Yvette, 91190 France; 3 Emergency Department, Saint Louis Hospital AP-HP, Clichy, France; 4 Emergency Department, Bichat Hospital AP-HP, Clichy, France; 5 Cardiology Department, Bichat Hospital AP-HP, Clichy, France; 6 INSERM UMR-S942, MASCOTT, Paris, France; 7 University of Paris Cité, Paris, France

**Keywords:** Electrocardiogram, Emergency physicians, Cardiologists, Abnormalities, Quality, Reading performance

## Abstract

**Background:**

Electrocardiogram (ECG) is one of the most commonly performed examinations in emergency medicine. The literature suggests that one-third of ECG interpretations contain errors and can lead to clinical adverse outcomes. The purpose of this study was to assess the quality of real-time ECG interpretation by senior emergency physicians compared to cardiologists and an ECG expert.

**Methods:**

This was a prospective study in two university emergency departments and one emergency medical service. All ECGs were performed and interpreted over five weeks by a senior emergency physician (EP) and then by a cardiologist using the same questionnaire. In case of mismatch between EP and the cardiologist our expert had the final word. The ratio of agreement between both interpretations and the kappa (k) coefficient characterizing the identification of major abnormalities defined the reading ability of the emergency physicians.

**Results:**

A total of 905 ECGs were analyzed, of which 705 (78%) resulted in a similar interpretation between emergency physicians and cardiologists/expert. However, the interpretations of emergency physicians and cardiologists for the identification of major abnormalities coincided in only 66% (k: 0.59 (95% confidence interval (CI): 0.54–0.65); *P*-value = 1.64e-92). ECGs were correctly classified by emergency physicians according to their emergency level in 82% of cases (k: 0.73 (95% CI: 0.70–0.77); *P*-value ≈ 0). Emergency physicians correctly recognized normal ECGs (sensitivity = 0.91).

**Conclusion:**

Our study suggested gaps in the identification of major abnormalities among emergency physicians. The initial and ongoing training of emergency physicians in ECG reading deserves to be improved.

**Supplementary Information:**

The online version contains supplementary material available at 10.1186/s12909-023-04670-x.

## Introduction

Electrocardiograms (ECGs) are routine exams in the emergency department (ED). ECG is a painless, noninvasive way to diagnose many acute heart diseases, but misinterpretation may lead to inappropriate care. Breen CJ et al. reported major errors in up to 33% of ECG interpretations, and up to 11% resulted in inappropriate care [1]. A meta-analysis by Cook et al. published in 2020 highlights deficiencies in ECG interpretation [2]. There is no such thing as an established standard method for “teaching” ECG interpretation [1, 3]. The development of new diagnostic tools, such as ECG interpretation algorithms, is undeniable, but various studies have shown their current limitations [4, 5]. Overreliance on artificial aid may also lead to inappropriate care [6]. ED doctors must be able to correctly analyze ECGs; most recently, many publications have assessed students’ or residents’ ECG reading capacity in the emergency department [2, 7–9] with or without training. In 2022, ECG interpretation competency among healthcare professionals and students was assessed [10]. However, few studies have assessed the ECG analysis capacity of senior emergency physicians [2], particularly in front of cardiologists who confirm or reject the initial interpretation in daily practice. This study aimed to assess the ability of ECG interpretation by emergency physicians compared to cardiologists and an ECG expert.

## Methods

### Study design and setting

This observational and prospective study was conducted in two emergency departments (Bichat University Hospital, Paris, and Beaujon University Hospital, Clichy) of two university hospitals and in one emergency medical service (Beaujon University Hospital, Clichy) over 6 weeks in 2019, between September 23 and October ^27^.

We included all 12- or 18-lead ECG performed in these three centers and interpreted during working hours day or night by senior emergency physicians who completed a questionnaire. We excluded all ECGs interpreted by juniors or medical students, all questionnaires without ECGs, or ECGs without questionnaires and ECGs considered uninterpretable by cardiologists.

There was a unique ECG per patient, and the questionnaire was anonymous, without epidemiological data and principally focusing on the reason for ED visit (appendix).

It was designed according to a protocol recently published [4]: it proposed 56 ECG abnormalities divided into 3 categories: urgent, significant and nonsignificant abnormalities.

The questionnaire followed the iterative thought process of an emergency physician who needs ECG to perform appropriate care for the patient. A question about its normality was first raised. If it was not normal, the emergency degree had to be mentioned as well as its significant characteristics.

We defined “Urgent” abnormalities requiring extreme urgent care; “Significant” abnormalities requiring serious consideration and relative emergency; “non-significant” minor or non-specific abnormalities; and “Normal” ECG without abnormality including normal variant. All ECGs were numbered and then separated from the questionnaire completed by emergency physicians.

An identical empty questionnaire was attached to each ECG and had been randomly distributed to 16 senior cardiologists of Bichat hospital. They performed a second analysis of the ECGs and completed the questionnaire unaware of the first analysis. Only the reason for ED consultation was known by cardiologists.

If there was a discordant interpretation between the emergency physician and cardiologist, we sought the opinion of an ECG expert (Dr Pierre Taboulet). The expert gave his interpretation independently, and the latter was considered the reference. If the expert’s interpretation was the same as that of the cardiologist, it was considered that there was a consensus between the cardiologist and the expert. If the expert’s interpretation differed from that of the cardiologist, he would reread the ECG, having taken note of the interpretations of both the emergency physician and the cardiologist, in order to reach a consensus after discussion. Finally, if no consensus was made for an ECG, it was decided to arbitrarily exclude it from this study.

### Statistical analysis

The primary endpoint was to assess the quality of ECG interpretation by emergency physicians, defined as the level of concordance to those performed by cardiologists/experts for major anomaly recognition (urgent and significant). This was assessed by the ratio of agreement and Cohen’s kappa coefficient (k) with their 95% confidence interval (CI) [11, 12]. The ECG reading quality was respectively qualified as excellent, good, average, or poor if ≥ 90, ≥ 80, ≥70, ≤ 70% of the major abnormalities were correctly identified by the emergency physicians.

The kappa coefficient measures the interpretive agreement between the two raters.

We defined:

- Perfect agreement 0.8 < kappa < 1.
- Strong agreement 0.6 < kappa < 0.8.
- Moderate agreement 0.4 < kappa < 0.6.
- Weak agreement 0.2 < kappa < 0.4.
- Very weak agreement 0 < kappa < 0.2.

As a secondary endpoint, we observed the performance of emergency physicians in classifying an electrocardiogram according to its level of emergency. Specificity (Sp), sensitivity (Se), positive predictive value and negative predictive value with their 95% CI were also calculated.

Data analyses were performed using R version 4.2.2. All statistical tests were two tailed, and a p-value of less than 0.05 was considered statistically significant.

## Results

**Fig. 1:**
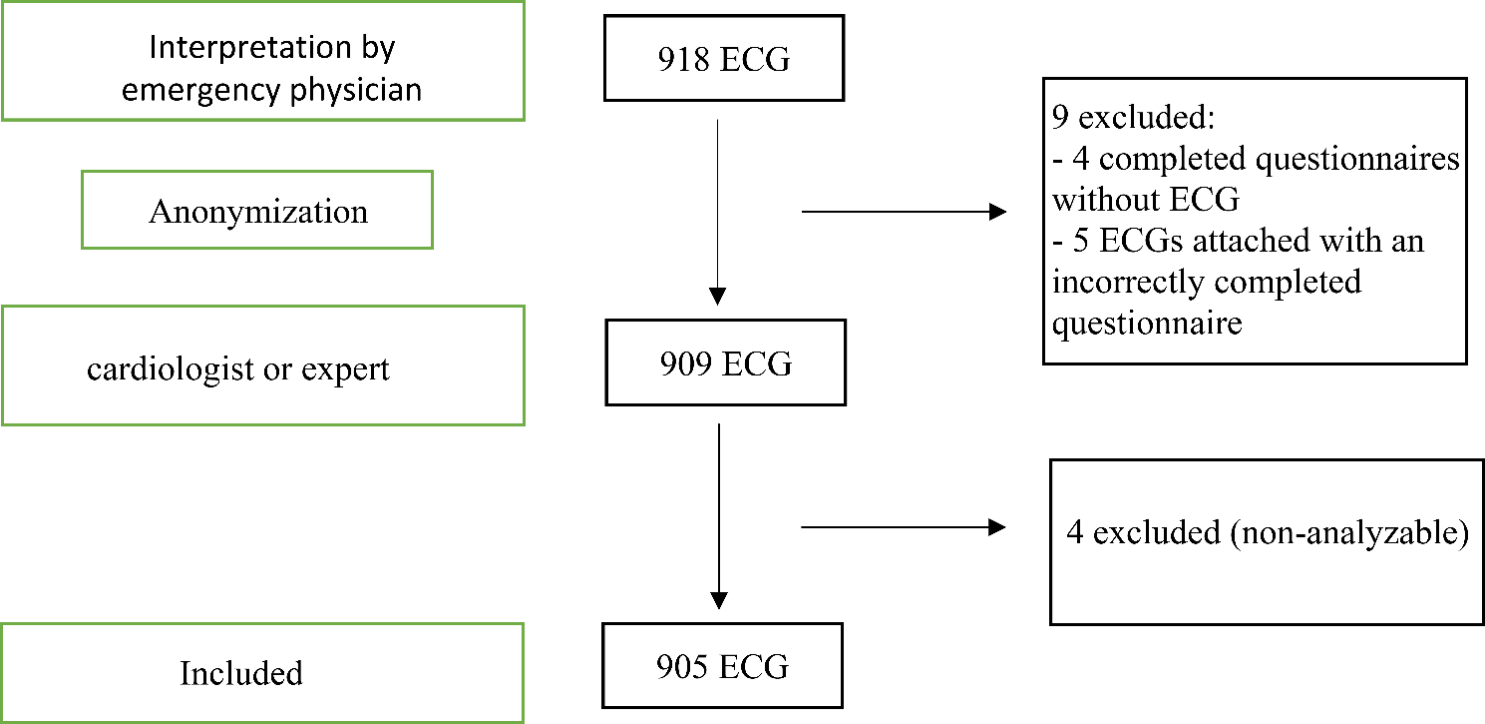
Flow chart

We collected 918 ECGs and analyzed 905 ECGs (Fig. 1).

Nine were excluded, five by emergency physicians because questionnaires were not correctly completed or not attached to the right ECG, and four by cardiologists and/or experts because they considered them uninterpretable (artefacts, errors due to electrode position and one to pathological issues due to resuscitation after cardiac arrest). There were 296 discordant ECGs between emergency physicians and cardiologists. The expert reread all 296 ECGs. Among which he agreed with the cardiologists for 150 ECGs, with the emergency physicians for 81 ECGs and with neither for the remaining 65.

**Fig. 2:**
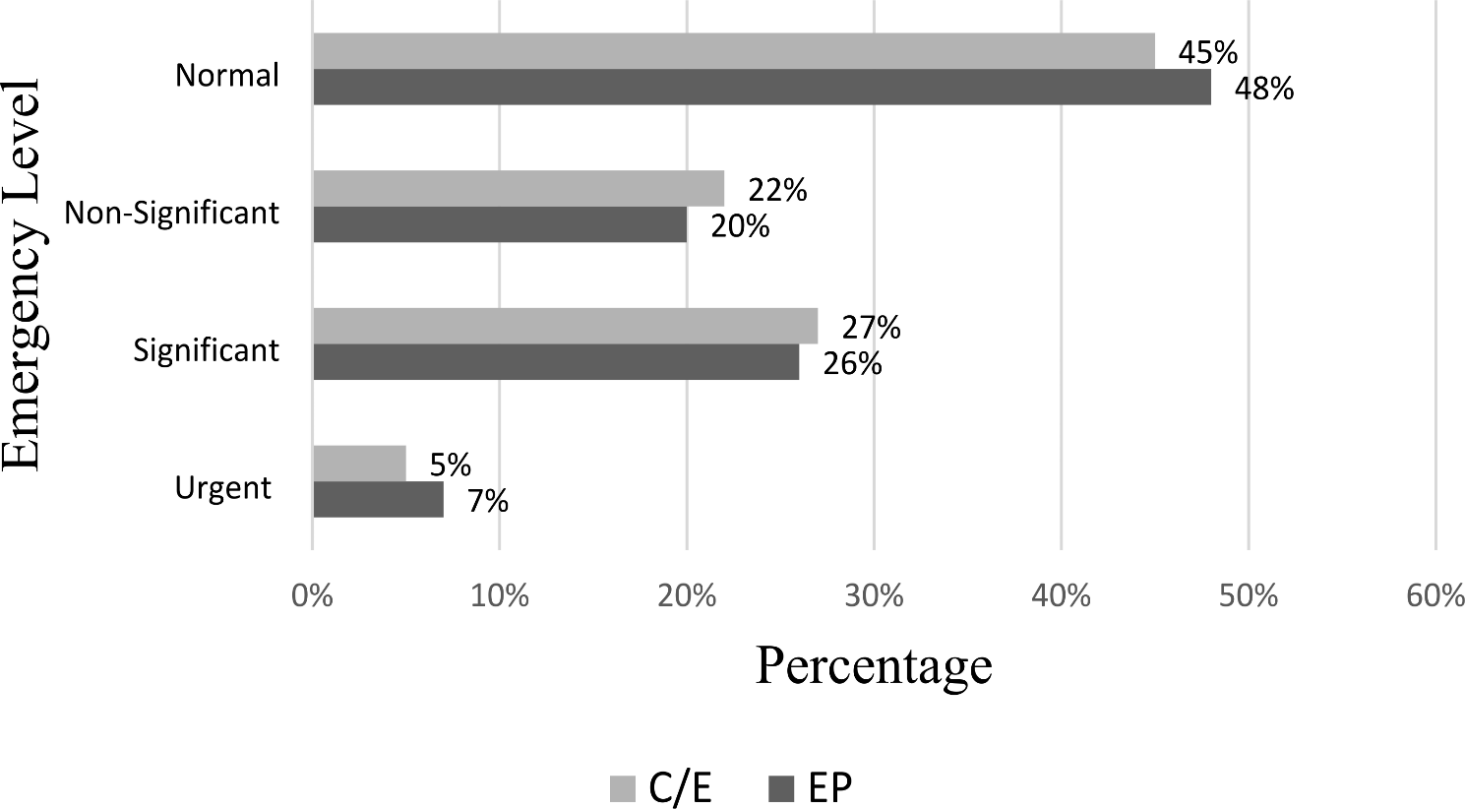
Distribution of interpreted ECGs according to their emergency level (C/E: cardiologists and experts; EP: emergency physician)

We reported 63 different reasons for consultation, and the most common were chest pain (16%), discomfort (8%), abdominal pain (6%), dyspnea (3%), fall (3%) and palpitations (3%). The reason was not mentioned for 319 ECGs (35%). Cardiologists and experts (C/E) classified 49 urgent (5%), 246 significant (27%), 200 nonsignificant (22%) and 410 (45%) normal ECGs. Emergency physicians listed 61 urgent (7%), 232 significant (26%), 178 non-significant (20%) and 434 (48%) normal ECGs (Fig. 2).

The most common urgent anomalies (Table 1) detected by C/E were atrial fibrillation with a heart rate greater than 120 bpm (29%), NSTEMI (non-ST elevation myocardial infarction) (22%) and STEMI (ST elevation myocardial infarction) (19%).

**Table 1:** Urgent anomalies recognized by cardiologists/experts

URGENT Anomalies	N (%)
*Atrial Fibrillation Heart Rate > 120 bpm*	14 (29%)
*N-STEMI*	11 (22%)
*STEMI*	9 (19%)
*Junctional tachycardia*	5 (10%)
*Ventricular tachycardia*	2 (4%)
*Bradycardia < 45 bpm*	2 (4%)
*Atrial Flutter*	1 (2%)
*Pacemaker Rhythms > 120 bpm*	1 (2%)
*Third-degree atrioventricular block*	1 (2%)
*Hyperkalemia*	1 (2%)
*Pericarditis*	1 (2%)
*Long QT syndrome > 500 ms*	1 (2%)
***TOTAL***	**49** (100%)

ms: millisecond; bpm: beat per minute; N-STEMI: non-ST Elevation Myocardial Infarction, STEMI: ST Elevation Myocardial Infarction

The most common significant anomalies detected by C/E (Table 2) were tachycardia with a heart rate above 100 bpm (28%), complete right bundle branch block (14%), atrial fibrillation with a heart rate between 45 and 120 bpm (12%), rhythm driven by a pacemaker between 45 and 120 bpm (9%), chronic STEMI sequels (8%) and ventricular hypertrophy (8%). ECG interpretations by emergency physicians matched those by C/E in 78% of cases.

**Table 2:** Significant anomalies recognized by cardiologists/experts

SIGNIFICANT ANOMALIES	N (%)
*Sinus tachycardia > 100 bpm*	80 (28%)
*Right Bundle Branch Block*	40 (14%)
*Atrial Fibrillation Heart Rate between 45 et 120 bpm*	34 (12%)
*Pacemaker Rhythms between 45 et 120 bpm*	26 (9%)
*Old myocardial infarction*	25 (9%)
*Ventricular hypertrophy*	23 (8%)
*Repolarization suggestive of myocardial ischaemia*	17 (6%)
*Left Bundle Branch Block*	13 (5%)
*Atrial and ventricular extrasystoles*	11 (4%)
*Long QT syndrome between 470 et 500ms*	5 (2%)
*Pre-excitation syndromes*	3 (1%)
*Sinus bradycardia < 45 bpm*	2 (0,7%)
*Supraventricular non-sinus rhythm between 45 and 120 bpm*	1 (0,4%)
*Brugada ECG*	1 (0,4%)
**TOTAL**	**281** (100%)

ms: millisecond; bpm: beat per minute; N-STEMI: non-ST elevation myocardial infarction, STEMI: ST elevation myocardial infarction

Primary endpoint:

In 66% of cases, emergency physicians and cardiologists/experts identified the same major (urgent and significant) abnormalities for 196 (38+158) ECGs out of 295 (49+246). The Kappa coefficient (k) was 0.59 (95% confidence interval (CI): 0.54–0.65); *P*-value = 1.64e-92 (Table 3).

Among the 49 ECGs classified as urgent by the cardiologists/experts, 38 (78%) were correctly interpreted by the emergency physicians (k = 0.69 (95% CI: 0.59–0.79); *P*-value = 3.5e-40).

Among the 246 ECGs showing the most serious abnormalities recognized by the cardiologists/experts and classified as significant, 158 (64%) were correctly interpreted by the emergency physicians (k = 0.62 (95% CI: 0.57–0.69); *P*-value = 1.65e-96)).

**Table 3:** Table of concordance of recognition of abnormalities present on ECGs between emergency physicians and cardiologists/experts

	CARDIOLOGIST/EXPERT					
EMERGENCY PHYSICIAN		**1**	**2**	**3**	**4**	TOTAL
	**1**	**38**	10	8	2	**58**
	**1≠**	3				3
	**2**	5	**158**	17	15	**195**
	**2≠**		37			37
	**3**	0	22	**136**	20	**178**
	**4**	3	19	39	**373**	**434**
	TOTAL	**49**	**246**	**200**	**410**	**905**
	**CONCORDANCE**	**78%**	**64%**	**68%**	**91%**	**78%**
	**Kappa**	**0,69**	**0,62**	**0,64**	**0,78**	

**1**: Urgent ECG

**1**≠: urgent ECG whose abnormality(s) recognized by the emergency physician is (are) urgent but different from that(s) recognized by the cardiologist/expert.

**2**: significant ECG.

**2**≠:significant ECG whose abnormality(s) recognized by the emergency physician is (are) significant but different from that(s) recognized by the cardiologist/expert.

**3**:ECG not significant.

**4**:normal ECG.

**Concordance**: percentage of concordance of ECG interpretation between emergency physician and cardiologist/experts by degree of emergency.

**Kappa**: degree of agreement in ECG interpretation between cardiologists/experts and emergency physicians.

Secondary endpoint:

ECGs were consistently classified in 82% of the cases on the degree of urgency (without looking at the diagnosis accuracy) between cardiologists/experts and emergency physicians (Table 4). Namely, emergency physicians identified 41 out of 49 ECGs (84%) classified as “urgent” by cardiologists/experts, and similarly, 195 out of 246 ECGs (79%) classified as “significant”, 136 out of 200 ECGs (68%) classified as “non significant”, and 373 out of 410 ECGs (91%) classified as normal by cardiologists/experts.

The Kappa coefficient was 0.73 (0.70–0.77); *P*-value ≈ 0. The weighted Kappa was 0.78 (74–0.81) with linear weighting and 0.81 (0.81–0.81) with quadratic weighting (Table 4).

The ability of the emergency physicians to discerning normal ECGs (91%) was globally good but less accurate for “urgent” (85%), “significant” (79%) and “non-significant” (68%) ECGs.

**Table 4:** Concordance table of ECG classifications according to the degree of urgency between emergency physicians and cardiologists/experts

		CARDIOLOGIST/EXPERT				
EMERGENCY PHYSICIAN		**1**	**2**	**3**	**4**	Total
	**1**	**41**	10	8	2	**61**
	**2**	5	**195**	17	15	**232**
	**3**	0	22	**136**	20	**178**
	**4**	3	19	39	**373**	**434**
	Total	**49**	**246**	**200**	**410**	**905**
	Concordance	**83,7%**	**79,3%**	**68%**	**91%**	**82,3%**
	Weighting	None	Linear	Quadratic		
	Kappa	0.73	0.78	0.81		

**1**: “Urgent” ECG.

**2**: “significant” ECG.

**3**: “non-significant” ECG.

**4**: Normal ECG.

**Equal**: weights are linear because they are proportional to the difference between two assessments of an individual.

**Square**: the weights are quadratic because they are proportional to the square of the difference between two assessments.

However, the specificity of recognition of normal ECGs (88%) was not better than that of “urgent” (98%), “significant” (94%) and “non-significant” (94%) ECGs (Table 5).

The positive predictive value was better for normal ECGs (86%), then in decreasing order for significant (84%), non-significant (76%) and urgent (67%) ECGs. In contrast, the negative predictive value was better for urgent ECGs (99%) than for normal (92%), significant (92%) and non-significant (91%) ECGs.

Regarding the three most frequent emergency abnormalities present on ECGs, atrial fibrillation with a heart rate greater than 120 bpm seemed to be moderately recognized by emergency physicians (Se = 0.79). Meanwhile, they appeared to be very good at identifying ECGs compatible with ST- coronary syndrome (Se = 0.91) and ST + coronary syndrome (Se = 1) (Table 5).

**Table 5:** Sensitivity, specificity, positive predictive value and negative predictive value of the recognition of the ECG urgency level and the three most frequent abnormalities by emergency physicians

ECG urgency level	Se (95% CI)	Sp (95% CI)	PPV (95% CI)	NPV (95% CI)
URGENT	0.84 (0.70–0.93)	0.98 (0.96–0.99)	0.67 (0.54–0.79)	0.99 (0.98- 1.00)
SIGNIFICANT	0.79 (0.74–0.84)	0.94 (0.92–0.96)	0.84 (0.79–0.89	0.92 (0.90–0.94)
NON SIGNIFICANT	0.68 (0.61–0.74)	0.94 (0.92–0.96)	0.76 (0.69–0.82)	0.91 (0.89–0.93)
NORMAL	0.91 (0.88–0.94)	0.88 (0.84–0.90)	0.86 (0.82–0.89)	0.92 (0.89–0.94)
**Abnormalities**	**Se (95% CI)**	**Sp (95% CI)**	**PPV (95% CI)**	**NPV (95% CI)**
AF (HR > 120 bpm)	0.79 (0.49–0.95)	1 (0.99-1.00)	0–92 (0.62- 1.00)	1 (0.99-1.00)
N-STEMI	0.91 (0.59-1.00)	0.99 (0.98-1.00)	0.59(0.33–0.82)	1 (0.99-1.00)
STEMI	1 (0.66-1.00)	0.99 (0.99-1.00)	0.60 (0.32–0.84)	1.00 (1.00–1.00)

***Se***: sensitivity, ***Sp***: specificity, ***PPV***: positive predictive value, ***NPV***: negative predictive value, 95% CI: 95% confidence interval, AF: atrial fibrillation, N-STEMI: non-ST elevation myocardial infarction, STEMI: ST elevation myocardial infarction

## Discussion

Emergency physicians showed good capacity to correctly identify major abnormalities. The recognition accuracy of major anomalies present on an ECG was critical because these anomalies might an impact patient management. Similarly, discordant classification between a normal ECG and a “non-significant” ECG could not harm the patient. Kappa coefficients were calculated to support these results, which was a measure of inter-rater agreement. The agreement between cardiologists/experts and emergency physicians was moderate for major abnormalities.

The overall agreement in the interpretation of ECGs between emergency physicians and cardiologists/experts is much better than the agreement in the interpretation of major abnormalities. This could be explained by the fact that a large proportion of the ECGs included in the study were normal and that the ability to identify them by emergency physicians was excellent. The results can be generalized to the French population consulting the emergency department, notably because of the large sample size but also because the reasons leading to the consultation during which the ECGs were performed in our study were similar to the reasons for consultation identified in the other studies [13]. Additionally, the high proportion of normal ECGs and the most frequently recognized abnormalities were similar to other studies [4, 13].

Beyond discerning the correct abnormality, it is more important to correctly classify an ECG according to its emergency level. This is mainly because “urgent” ECGs require rapid management, and “significant” ECGs have clinical significance for the diagnostician and may lead to additional investigations or advice from a cardiologist.

In general, ECG classification according to their degree of urgency by emergency physicians seemed good, but emergency physicians tended to recognize more urgent abnormalities than C/E. This could lead to overmedicalization, which could be harmful to the patient. They also had more difficulties to recognize nonsignificant abnormalities. The Kappa linear weighting and quadratic weighting were higher than the unweighted Kappa coefficient for the classification of the different ECGs. This means that the consistency of ECG classification between the two evaluators (emergency physicians vs. cardiologists/experts) appears to be higher when the clinical significance of the abnormality is taken into account.

The sensitivity of the emergency physicians concerning the recognition of normal ECGs was globally good but less efficient for other stages of emergency. On the other hand, the specificity of recognition of normal ECGs was worse than that of the others. In other words, emergency physicians recognized normal ECGs well but abnormalities too often. Moreover, when they considered an ECG as “non urgent”, the probability that this ECG was indeed not an emergency was very high.

Among the three most frequent emergency abnormalities present on ECGs performed in the emergency department, atrial fibrillation with a heart rate greater than 120 bpm was moderately recognized by emergency physicians. While, they were very good at matching ECGs compatible with STEMI and N-STEMI.

Our results were consistent with those in the literature [1]. ECG interpretation proved to be a difficult exercise [1, 2]. Some studies have shown that noncardiologists make more ECG interpretation errors than cardiologists [2, 10]. Interpretation algorithms can reduce the time needed to interpret ECGs and can reduce ECG interpretation errors [1]. However, they have been shown to be less accurate than physicians and should only be used as an additional interpretation tool for a trained provider [1, 3]. While continuing education of emergency physicians in ECG analysis is a major issue [14, 15], evidence of the need for universal predefined training to achieve and maintain ECG interpretation skills is not available [1]. Several methods could be used, such as simulation [16], self-study [17] or work-shops [18].

The implementation of an ECG reading checklist in emergency departments could possibly decrease the rate of ECG misinterpretation. A double reading of emergency ECGs by an ECG expert could be considered to catch potential misdiagnoses. The development of an interactive interpretation aid application with a training function could meet the need for continuing education of residents.

### Limitations

The study was a multicentric study, but it concerns 3 centers in Ile de France that are geographically close. Moreover, the vast majority of the doctors practicing in the EMS also work in one of the emergency departments. A multicentric study integrating several emergency services spread all over France would truly represent the performances of French emergency physicians concerning the interpretation of ECG.

Clinical experience increased ECG interpretation competency [19, 20]. In the present study, the population of emergency physicians who answered the questionnaire was not analyzed: training and number of years as a senior.

The study shows that the ability of emergency physicians to identify major anomalies is poor. However, the interpretation of an ECG is always carried out in a precise clinical context (age, history, symptomatology and clinical examination) with blood tests and possible advice from the cardiologist.

It should be noted that the cardiologists did not have this information. The absence of a global context makes the interpretation of ECGs more difficult for them.

We chose cardiologists and experts as the reference for ECG interpretation in our study because it has been proven that cardiologists are the best for ECG interpretation. However, the literature indicates that the accuracy of ECG interpretation by cardiologists was 74.9% [2]. As this was not perfect, choosing cardiologists as the reference for interpretation represents a bias. Double analysis by the cardiologists and by the expert allowed us to limit this bias.

Our consecutive cases study assessed both specificity (consecutive cases have a large number of normal ECGs). But sensitivity is also important, and this requires a large number of true positives. The methods for such studies could be a combination of case control and consecutive in order to have lots of normal and lots of abnormal.

## Conclusion

The overall interpretation accuracy of ECGs in our study seems to be good, and emergency physicians seem to be quite good at determining the degree of urgency of an ECG. However, their reading of major anomalies is poor compared to cardiologists.

The initial and ongoing training of emergency physicians in ECG reading should be improved.

### Electronic supplementary material

Below is the link to the electronic supplementary material.

Supplementary Material 1

## Data Availability

The datasets used and/or analysed during the current study are available from the corresponding author on reasonable request.

## Abbreviations

CI: Confidence Interval
ECG: Electrocardiogram
ED: Emergency Department
EP: Emergency Physician
Se: Sensitivity
Sp: Specificity
