# Supplementary material for: Assessment of real-time electrocardiogram effects on interpretation quality by emergency physicians
Source: BMC Med Educ. 2023 Sep 18;23:677. doi: 10.1186/s12909-023-04670-x (PMC10506301; doi:10.1186/s12909-023-04670-x)
Supplement: Supplementary file 1 — Supplementary Material 1 [file 12909_2023_4670_MOESM1_ESM.docx]

**Appendix: ECG interpretation questionnaire**

1. Is this ECG normal? □ Yes □ No

If Yes, Stop here.

2. If Not, Is this ECG urgent? □ Yes □ No

If yes, tick the corresponding urgent anomaly: (If Not, skip to question 3)

Rhythm emergencies

□ Sino-auricular block

□ Atrial Fibrillation with HR > 120

□ Atrial Fibrillation with HR < 45 bpm

□ Supra-ventricular non-sinus tachycardia (HR > 120 bpm)

□ Atrial flutter

□ Focal atrial tachycardia (FAT)

□ Multi-focal atrial tachycardia

□ Intranodal junctional reentry tachycardia

□ Junctional accessory pathway reentry tachycardia

□ Supra-ventricular non-sinus bradycardia (HR < 45 bpm)

□ Pace trained rhythm > 120bpm

□ Pace trained rhythm < 45bpm

Ventricular rhythm emergency

□ Ventricular Tachycardia

□ Ventricular fibrillation

□ Ventricular escape rhythm HR < 45 bpm

□ Indefinite rhythm

Atrioventricular conduction emergency

□ Second-degree Atrioventricular heart block Mobitz Il

□ Third-degree Atrioventricular heart block

Other emergency

□ Hyperkalemia

□ Hypokalemia

□ Hypothermia

□ STEMI or Recent Myocardial infraction

□ N-STEMI

□ Myocarditis / Pericarditis

Repolarization emergency

□ Long qt syndrome > 500ms

3. If Not, Is this ECG present one or more significant anomalies: □ Yes □ No

Rhythmic abnormalities

□ Sinus bradycardia < 45 bpm

□ Sinus tachycardia > 100 bpm

□ Atrial Fibrillation between 45 et 120 bpm

□ No sinus supra-ventricular rhythm entre 45 et 120 bpm

□ Pace trained rhythm 45 <HR <120

□ Ventricular rhythm 45< HR 120

Atrioventricular conduction abnormalities

□ Second-degree Atrioventricular heart block Mobitz I

□ Pre-excitation

□ Ventricular extrasystoles bursts or Atrial extrasystoles in couplets, triplets, bigeminism, trigeminism

QRS complex abnormalities

□ Complete right limb block

□ Complete left limb block

□ Ventricular Hypertrophy

ST segment and repolarization abnormalities

□ Old myocardial infraction

□ Ventricular Repolarization suggestive of Myocardial Ischemia

□ QT interval between 470 and 500ms

□ U Wave

□ Short QT interval <320ms

Other

Rare pathologies (Brugada...)

4. If Not, Is there minor abnormalities on this ECG: □ Yes □ No

Minor abnormalities: Isolate Ventricular extrasystoles, Isolate Atrial extrasystoles, First-degree Atrioventricular heart block, Atrial hypertrophy, right axis, left axis, fascicular bloc, incomplete right limb block, Nonspecific repolarization abnormalities, Borderline QT interval (between 430 et 470ms), microvoltage.

(ECG: electrocardiogramme; HR: heart rate; bpm: beat per minute
